# Bone health - An investment

**DOI:** 10.4103/0019-5413.53450

**Published:** 2009

**Authors:** Anil K Jain, Surender Singh Yadav

**Affiliations:** *Editor, Indian Journal of Orthopaedics, Prof. of Orthopaedics in University College of Medical Science, Senior Orthopaedic Surgeon GTB Hospital, Delhi, India*; 1 *Ex. Director, Pt. BD Sharma Institute of Medical Sciences (Rohtak), Past president Indian Orthopaedic Association and Chairman, Ram-Bhagwan Charitable cancer hospital trust, Mirpur, Haryana, India*

The skeleton provides support, mobility and protection for human body and acts as a reservoir to store essential minerals. Bony mass and its architecture or shape to provide adequate strength and mobility are determined by the mechanical forces that act on the skeleton. Each species has a genetically determined shape, size and volume of bone that is constantly modified during life time in response to mechanical stresses applied to the bones.

Bone is a composite material consisting of crystals of mineral bound to protein. The calcium and phosphate in the shape of a hydroxyapetite crystal is bound in an orderly manner to a matrix made up of protein called collagen. Thus the bone formed is strong, resilient and not brittle. Bones as a part of the musculoskeletal system provide lever for muscles to move the limb. The mechanical forces provided by muscle action constantly put stresses to the bone to maintain bone mass. The bone mass and strength is reduced considerably if the bone is not mechanically loaded as in paralytic conditions such as post polio residual paralysis, muscular dystrophies or when the limb is in plaster or during long duration space travel.

The bone is a dynamic tissue. The bone continues to undergo modeling and remodeling throughout life. In pediatric age group, bone undergoes modeling where new bone is formed at one site with removal at another site in the same bone. Thus individual bones grow in size, length, width and volume size. Around puberty the bone gets thicker. The size and shape of bone are genetically determined but can be greatly influenced by the loading or impact that occurs with physical activity.^1^ To develop a good bone volume the body requires good nutrition including proteins, minerals, vitamin D along with physical activity. Children getting good nutritious diet and participating in sports develop better bone mass. The bone mass continues to increase in childhood and attains a peak bone mass between 20-25 years. After attaining peak bone mass the human bones loose one per cent bone every year. Females start losing three per cent bone every year after menopause while men continue to loose one per cent per year. After 70 years men also loose bone as rapidly as women. If a person attains a good peak bone mass he/she is likely to maintain good bone volume at 70 years or more. It is just like starting with a good bank balance and losing money only at a certain rate per year. Someone with better bank balance continues to live without exhausting total bank balance.

The bone undergoes constant remodeling throughout life where a small amount of bone on the surface of trabeculae or in the interior of the cortex is removed and replaced at the same site. This process helps in repair of the damage to the skeleton and replaces the accumulated, very old bone (may have lost its resilience and become brittle). The resorption of bone on the surface of trabecular bone supplies calcium and phosphorus to maintain their blood levels for various essential functions such as clotting mechanism, muscle contraction, for the fetus and during lactation. A complex hormonal mechanism assists in this function. These processes continue through out life and most adult skeleton is replaced about every 10 years.

In short, both genes and environment contribute towards bone health. Genes determine the size and shape of the skeleton and environmental factors such as diet and physical activity determine the volume of bone mass. Nutritional deficiency, hormonal disorders lack of exercise, immobilization and smoking can lead to poor development of bone and accelerate the loss of bone mass.

Reduction in bone mass produces osteopenia and osteoporosis which predisposes bone to fracture. While other major organ diseases such as cancer or cardiac disease or cerebral strokes make newspaper headlines, fractures due to bone fragility do not get such attention as they do not cause instantaneous death. However, they leave a serious impact on the nation's health status in view of significant pain and height loss, costly treatment, residual disability, loss of man-day, handicap and even long run life risk due to complications such as pressure sores, pneumonia, and urinary tract infection. Once a fracture occurs in an osteoporotic skeletal, the chance of refracture increases. This affects the self esteem and creates fear psychosis. Data for average bone mass or burden of disease caused by fracture in osteoporotic population is not available for developing countries. One can make a rough estimate from the available data from developed countries. Over 10 million individuals over the age of 50 have osteoporosis in US and 33.6 million individuals have osteopenia.^2^ The US has a more aging population than India though the population of India is almost four times that of the US. The life expectancy of India has also crossed 65 years. The percentage of population having osteoporosis and osteopenia should be more in India in view of 70% population living below poverty line and never attaining good peak bone mass consequently developing osteopenia and osteoporosis earlier than US. Further increase in life expectancy will add more number to osteopenia and osteoporotic population. India has an abundance of undernourished children. Being a male dominated society, females get poor nutrition in childhood and even after marriage. Undernourished females go through multiple pregnancies to eventually become osteopenic and osteoporotic very early. Thus the figure of osteoporotic and osteopenic fractures may be many times more than developed countries. The urban society also has low Calcium and vitamin D intake in view of the reduced consumption of dairy products and increase in intake of fast food along with sedentary life, smoking and alcohol abuse.

Most of the problems described above could be prevented, if we consider bone health as important as cardiac health. Osteoporosis in the elderly is a result of the cumulative impact of bone loss and determination of bone structure.^3^ Various diseases such as vitamin D deficiency (nutritional or renal causes) in children and adults, chronic renal diseases, hormonal disorders and drug induced reduction in bone mass and need early investigation. A small change such as 10% increase in bone mass can reduce fracture risk by 50%.^4^ Good nutrition and physical activity are important for bone health for growth of skeleton in childhood and maintenance of skeleton in adults. The general calcium recruitment is 1300 mg/day between 9-18 of age, 1000 mg/day between 19-50 years and it increases to 1200 mg/day to those above 50 years. Similarly, vitamin D intake may vary from 200 IU/day up to 50 years to 400 IU/day between 50-70 years and 600 IU/day above 70 years.

Physical activity is important for bone health throughout life. During childhood it helps increase bone size and volume. The loading exercises before puberty increase bone size and bone resistance to bending.^5^ Most people have three to five per cent difference in their bone mass in dominant and non-dominant arm. This is an indirect indicator of the influence of physical activity on bone mass. Hence, increased physical activity during childhood helps increase bone mass, while during adulthood maintains bone mass. The aerobic weight bearing and resistance exercises were all effective in increasing bone mineral density (BMD) at the spine while walking was affective in increasing BMD at both hip and spine.^6^

This problem has more of a preventive strategy than treatment. We need to sensitize our rural and urban population about the importance of bone health. Bone health can be improved with education about the need for a balanced nutritious diet including dairy products, for every child, irrespective of gender. Playing should be an important constituent of curriculum particularly in adolescence and around puberty for all male and female children. Even after attaining adulthood one needs to continue with physical activity during the daily routine. At least 30 minutes of brisk walk everyday helps maintain bone mass. Doctors responsible for basic health needs in primary health centers or sub centers should leave no opportunity to increase awareness regarding bone health. Schools are the best place to educate children, our future citizens, about the concept of bone health and the role of lifestyle, nutrition in developing good peak bone mass and consequently preventing fragility fractures.

Studies should be conducted at a national level to quantify the status of bone health of our population, their level of awareness and steps to improve bone health. This small investment will pay rich dividends in the long run by preventing the occurrence of fragility fractures and improving the bone health and quality of life.
